# First Asian population study of stereotactic body radiation therapy for ventricular arrhythmias

**DOI:** 10.1038/s41598-021-89857-2

**Published:** 2021-05-14

**Authors:** Li-Ting Ho, Jenny Ling-Yu Chen, Hsing-Min Chan, Yu-Cheng Huang, Mao-Yuan Su, Sung-Hsin Kuo, Yeun-Chung Chang, Jiunn-Lee Lin, Wen-Jone Chen, Wen-Jeng Lee, Lian-Yu Lin

**Affiliations:** 1 Division of Cardiology, Department of Internal Medicine, National Taiwan University College of Medicine and Hospital, No. 7, Chuang-Shan South Road, Taipei, 100 Taiwan; 2 Division of Radiation Oncology, Department of Oncology, National Taiwan University Hospital, No. 7, Chuang-Shan South Road, Taipei, 100 Taiwan; 3 Department of Radiology, National Taiwan University College of Medicine, No. 7, Chuang-Shan South Road, Taipei, 100 Taiwan; 4 Department of Medical Imaging, National Taiwan University College of Medicine and Hospital, No. 7, Chuang-Shan South Road, Taipei, 100 Taiwan; 5 Cardiovascular Center, Taipei Medical University Shuang Ho Hospital, No. 7, Chuang-Shan South Road, Taipei, 100 Taiwan; 6 Institute of Epidemiology and Preventive Medicine, College of Public Health, National Taiwan University, No. 7, Chuang-Shan South Road, Taipei, 100 Taiwan

**Keywords:** Cardiac device therapy, Interventional cardiology

## Abstract

We report the first Asian series on stereotactic body radiation (SBRT) for refractory ventricular arrhythmia (VA) in Taiwanese patients. Three-dimensional electroanatomic maps, delayed-enhancement magnetic resonance imaging (DE-MRI), and dual-energy computed tomography (CT) were used to identify scar substrates. The main target volume was treated with a single radiation dose of 25 Gy and the margin volume received 20 Gy using simultaneous integrated boost delivered by the Varian TrueBeam system. Efficacy was assessed according to VA events recorded by an implantable cardioverter-defibrillator (ICD) or a 24-h Holter recorder. Pre- and post-radiation therapy imaging studies were performed. From February 2019 to December 2019, seven patients (six men, one woman; mean age, 55 years) were enrolled and treated. One patient died of hepatic failure. In the remaining six patients, at a median follow-up of 14.5 months, the VA burden and ICD shocks significantly decreased (only one patient with one ICD shock after treatment). Increased intensity on DE-MRI might be associated with a lower risk for VA recurrence, whereas dual-energy CT had lower detection sensitivity. No acute or minimal late adverse events occurred. In patients with refractory VA, SBRT is associated with a marked reduction in VA burden and ICD shocks, and DE-MRI might be useful for monitoring treatment effects.

## Introduction

Severe ventricular arrhythmia (VA) is a leading cause of sudden cardiac death in patients with structural heart disease. In this patient population, implantable cardioverter-defibrillator (ICD) placement remains the treatment of choice for both the primary and secondary prevention of sudden cardiac death. However, ICD therapy, especially in the presence of electrical storm (defined as 3 or more sustained episodes of severe VA or appropriate shocks from an ICD within 24 h), is associated with excess mortality and morbidity regardless of whether the therapy is appropriate or not^1^. Although, compared with anti-arrhythmic therapy, catheter ablation of abnormal ventricular substrates has been demonstrated to be an effective and preferable strategy for the management of electrical storm, the outcomes are less than ideal, especially in patients with non-ischemic cardiomyopathy^2^.

Stereotactic body radiation therapy (SBRT) is a treatment modality in radiation oncology that emerged in the late 1990s and flourished in the last 20 years. SBRT has become the standard of care for inoperable early-stage non-small cell lung cancer and liver cancer^3,4^, and is considered a treatment option for oligometastases in the brain, lung, bone, liver, and adrenal glands^5^. In addition to cancer treatment, SBRT techniques targeting benign central nervous system diseases such as arteriovenous malformations, seizure foci, and trigeminal neuralgia have demonstrated promising results. The technical and clinical advances in SBRT include managing the respiratory motion of target areas in the thorax and liver with four-dimensional (4D) computed tomography (CT) and respiratory gating systems, defining safe radiation dose levels for critical organs, and setting training and quality assurance standards for radiation oncology clinics worldwide.

The precise delivery of very high radiation doses to the target tissues has made SBRT a promising alternative to catheter ablation, particularly for arrhythmogenic substrates that are difficult or impossible to access. Recently, SBRT has been successfully applied to patients with severe VA with failed or contraindicated catheter ablation^6,7^. The first reported case series with five patients who received non-invasive cardiac radiation for ventricular tachycardia (VT) showed the efficacy of cardiac radioablation for VT using SBRT^6^. The same group published the first prospective phase I/II trial of electrophysiology-guided non-invasive cardiac radioablation for VT (ENCORE VT study) in 19 patients, which showed that the procedure was safe, well tolerated, and able to markedly reduce the VT burden^7^. In this study, we report the first Asian series on the use of SBRT for refractory VA in a Taiwanese group of patients with various underlying cardiac diseases. Our report has several unique features. First, owing to evidence supporting that radiation dose to heart substructures is associated with non-cancer death after SBRT in lung cancer patients^8^, and the suggestion of the Task Group 101 of the American Association of Physicists in Medicine (AAPM TG101) that the maximal dose to the heart and pericardium should not exceed 22 Gy in a single fraction^9^, we prudently decided to use the simultaneous integrated boost (SIB) technique to treat the main target volume with a single radiation dose of 25 Gy and the margin volume with 20-Gy radiation. Second, we included patients with a variety of underlying cardiac diseases, including ischemic cardiomyopathy (ICM), dilated cardiomyopathy (DCM), hypertrophic cardiomyopathy (HCM), and arrhythmogenic right ventricular cardiomyopathy (ARVC). Third, pre- and post-therapy imaging studies were also performed and included in the analysis.

## Methods

### Study population

The study was approved by the institutional review board of the National Taiwan University Hospital Ethics Committee. All study procedures were performed in accordance with relevant guidelines/regulations, and informed consent was obtained from all patients and/or their legal guardians. This study included patients with treatment-refractory VA. Patients considered eligible were those with age ≥ 18 years, three or more episodes of sustained VTs in the last 3 months or monomorphic premature ventricular contractions (PVCs) > 20%, and failure of anti-arrhythmic medication treatment and one or more catheter ablation procedures (or a contraindication to catheter ablation). In all patients, three-dimensional (3D) electroanatomic maps of the ventricles were obtained and regions of the substrates responsible for the clinical VTs were outlined. The substrate was defined as a scar with voltage ≤ 0.5 mV (normal tissue > 1.5 mV) and late potentials or abnormal potentials. Activation mapping was used if clinical VTs were clinically tolerable. Pace mapping during sinus rhythm was also performed to assess the exit site of clinical VTs. All patients received a detailed explanation of the risks of treatment from both the attending electrophysiologist and the radiation oncologist, and provided written informed consent for the treatment.

### Procedural workflow

**Figure 1:**
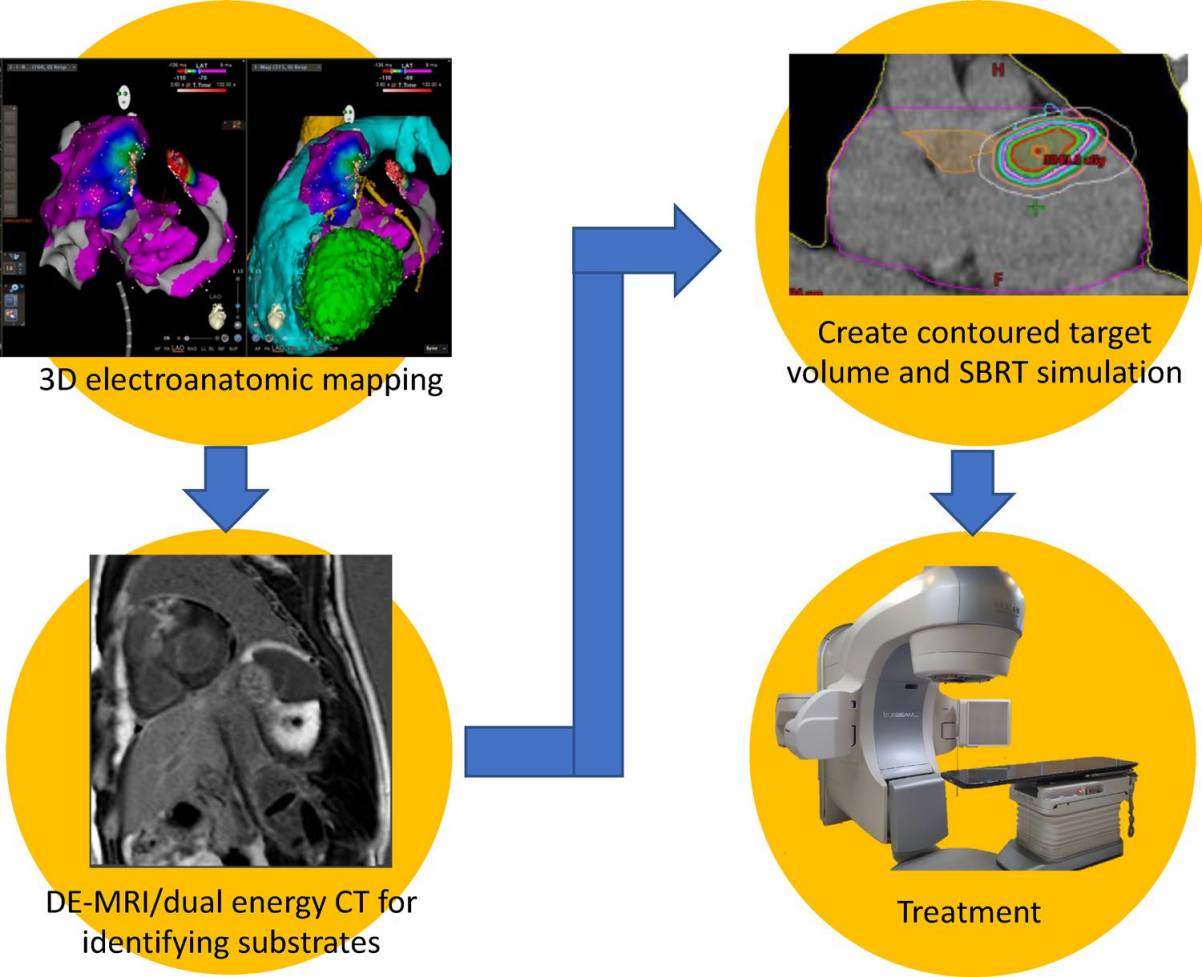
Workflow for stereotactic body radiation therapy of cardiac radioablation. The radioablation dose distribution figure was created by using treatment planning system (version 15.6, Eclipse, Varian Medical Systems, Palo Alto, URL: http://www.MyVarian.com).

The procedural workflow for SBRT is shown in Fig. 1. During the planning phase of the procedure, 3D electroanatomic maps were exported from the mapping system (CARTO-3; Biosense Webster, Diamond Bar, CA, USA) to a notebook with a viewer software. All patients underwent either electrocardiography (ECG)-gated delayed-enhancement magnetic resonance imaging (DE-MRI) or dual-energy CT to identify the scar regions. The CT/MRI images were visually compared with 3D electroanatomic maps, which showed the targets of SBRT. A contoured target volume was jointly created by the electrophysiologist, cardiac radiologist, and radiation oncologist. After an SBRT simulation, irradiation was performed and overseen by the radiation oncologist. The patients were hospitalized on the day of radiation treatment and discharged on the next day, in the absence of any immediate complication.

### Scar substrate identification

#### Cardiac magnetic resonance (CMR) imaging

CMR imaging was performed in patients with MRI-compatible ICDs. Late gadolinium enhancement (LGE)-MRI was performed 10 min after gadolinium injection to detect myocardial scarring. Because conventional LGE-MRI results in device-induced hyperintense artifacts that obscure ventricular images, wideband LGE-MRI was used to reduce the artifacts. The wideband LGE-MRI sequence was similar to the conventional sequence except for the bandwidth (inversion recovery [IR] sequence; repetition time/echo time, 6.7/3.2; resolution, 1.4 × 2.2 mm^2^; slice thickness, 8 mm; inversion time, 250–350 ms), which included a wideband IR radiofrequency pulse with frequency bandwidth and offset adjusted to minimize hyperintense artifacts. The default bandwidth of the wideband IR radiofrequency pulse was 3,800 Hz, and the optimal frequency offset was up to 8000 Hz^10,11^.

#### Dual-energy CT

All patients underwent ECG-gated dual-energy CT evaluation of myocardial scars before SBRT (Revolution HD; GE Healthcare, Milwaukee, WI, USA). ECG-gated non-contrast cardiac CT was performed first using prospectively gated axial scanning. Coronary CT angiography was subsequently performed using helical scanning with dose modulation. A multiphasic contrast injection protocol was used for better opacification of both the left- and right-sided heart chambers. The start of scanning was determined using a bolus tracking technique. Late iodine-enhancement CT was performed 7 min after coronary CT angiography.

Left ventricular scar tissue was defined as a region of wall thinning, hypoattenuation, decreased perfusion, or delayed enhancement on dual-energy CT or CMR imaging.

### Cardiac radioablation

All patients underwent a standard SBRT simulation while immobilized in an individualized vacuum bag (BodyFIX; Elekta, Stockholm, Sweden), which limited diaphragmatic motion through external abdominal compression. A free-breathing CT scan and a 4D-CT scan were acquired using a 16-slice CT scanner (Brilliance Big Bore CT; Philips Healthcare, Andover, MA, USA). Contrast was used during free-breathing CT to facilitate the definition of cardiac structures. Axial images (1-mm slices) were obtained. The treatment target volume was defined as described in “Procedural workflow”, and the location and shape of the target was outlined on the free-breathing CT scan using a treatment planning system (TPS) (Eclipse; Varian Medical Systems, Palo Alto, CA, USA). The target is referred to as the gross target volume (GTV). The internal target volume (ITV) was defined using 4D-CT to account for internal motion of the GTV caused by breathing and cardiac motion. ECG-gated CT was not performed because the device is not yet available at our institution; therefore, cardiac motion was measured integrated with respiratory motion in 4D-CT. The planning target volume (PTV) was defined as the ITV plus a 5-mm margin to account for any residual uncertainties in patient setup, motion, and delivery. Wherever the PTV overlapped with organs at risk (OARs), reducing the PTV to 3 mm at those slices was considered acceptable^12^. The dose constraints for single-fraction SBRT for OARs were adopted from the TG101, with the following maximal doses: skin, 26 Gy; rib, 30 Gy; main bronchus, 20 Gy; spinal cord, 14 Gy; stomach, 12.4 Gy; esophagus, 15.4 Gy; lungs (at least 1500 cm^3^ volume), < 7 Gy; liver (at least 700 cm^3^ volume), < 9.1 Gy. Because the TG101 suggested that the maximal dose to the heart and pericardium should not exceed 22 Gy in a single fraction, we set the maximal dose for the coronary artery (including the left anterior descending, right coronary, and circumflex arteries) as 22 Gy, and the heart volume excluding the PTV (heart minus PTV) receiving at least 16 Gy was kept at a < 15 cm^3^ value^9^.

**Figure 2:**
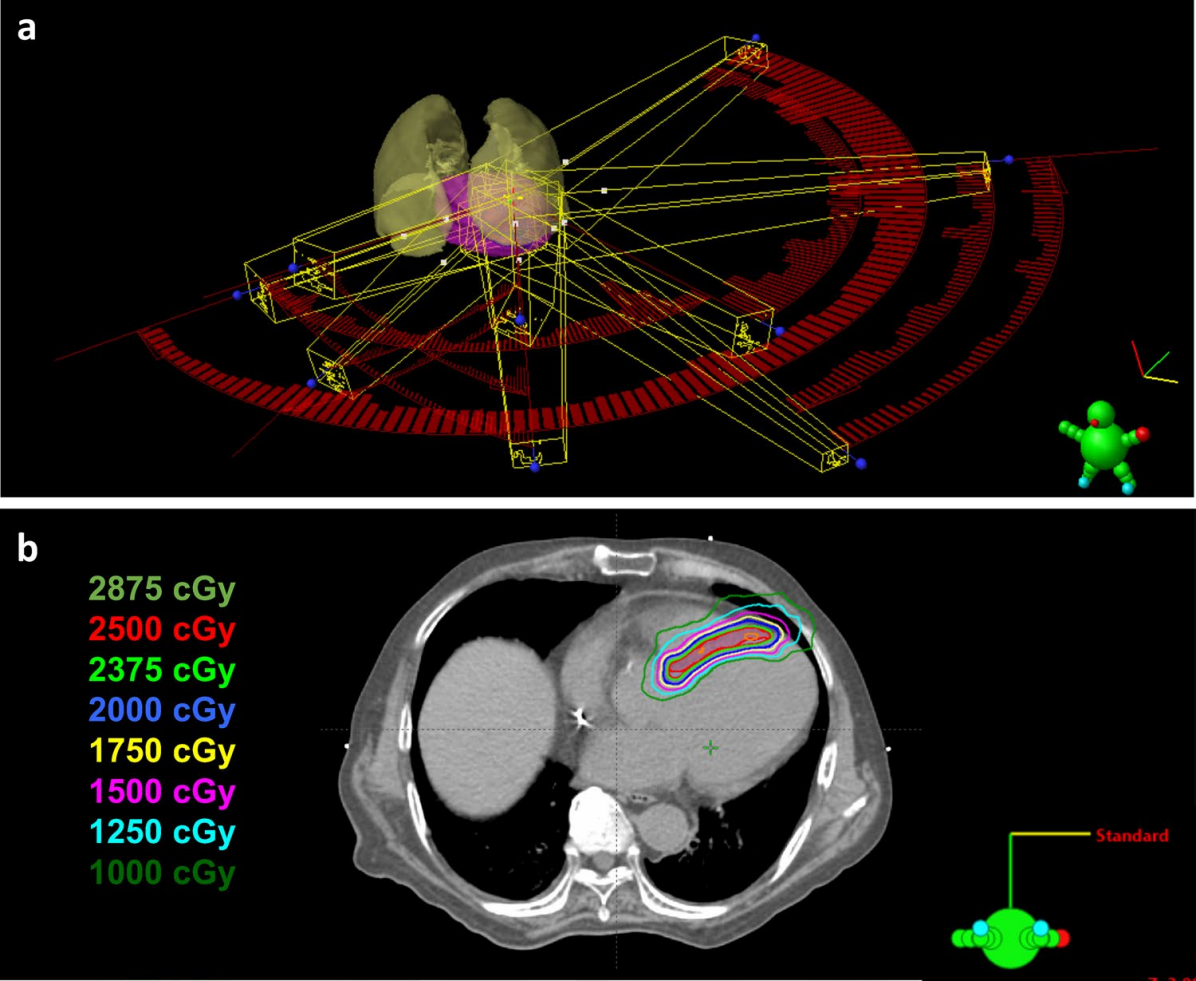
Noninvasive cardiac radioablation beam arrangement and isodose distributions. Patient 7 underwent noninvasive cardiac radioablation via 6 MV flattening filter-free photon beam and stereotactic volumetric modulated arc radiotherapy technique. A total of 25 Gy was prescribed to internal target volume (ITV) and 20 Gy to planning target volume (PTV) in a single fraction by simultaneous integrated boost strategy. Shown are (**a**) beam arrangement in noninvasive cardiac radioablation plan, and (**b**) dose distributions on the axial view. The colorwashed areas indicate the following: red, the ITV; and blue, the PTV. The orange, red, light green, blue, yellow, pink, indigo, and dark green lines represent isodose curves of 2875, 2500, 2375, 2000, 1750, 1500, 1250, and 1000 Gy, respectively. The radioablation dose distribution figure was created by using treatment planning system (version 15.6, Eclipse, Varian Medical Systems, Palo Alto, URL: http://www.MyVarian.com).

The non-invasive cardiac radioablation treatment plan was generated in the TPS using the SIB strategy to deliver 25 Gy to the ITV and 20 Gy to the PTV in a single fraction. Radiation dosimetry mandated that 95% of the target volume should receive the prescription dose (25 Gy for the ITV and 20 Gy for the PTV) and 99% of the target volume should receive a minimum of 90% of the prescription dose, and the maximal dose should be within the ITV up to 130% of the prescribed 25 Gy. A 6- or 10-MV flattening filter-free photon beam and volumetric-modulated arc radiotherapy technique were applied with multiple coplanar and non-coplanar ports, in which a highly accurate and conformal radiation dose was employed by adjusting the gantry rotation speed, dose rate, and shape of the multi-leaf collimator aperture. The orientation and direction of the radiation beams relative to the patient were selected with the goal of achieving maximal coverage of the target region while minimizing the dose to the surrounding OARs through the multiple coplanar and non-coplanar ports. The resultant plan was subjected to physics quality assurance a day before the procedure to ensure accurate dose delivery to the patient. An example of a non-invasive cardiac radioablation beam arrangement and isodose curves is shown in Fig. 2.

Irradiation was performed using an image-guided radiotherapy-equipped linear accelerator (Varian TrueBeam radiotherapy system; Varian, Palo Alto, CA, USA). The treatment position was verified and adjusted before and during each SBRT fraction using an onboard imaging device capable of acquiring volumetric images (cone beam CT [CBCT]). CBCT scans were first registered on the bony anatomy, followed by registration on soft tissue focused on the ITV, with refinements made by the treating radiation oncologist. Second, CBCT scans were acquired after correction, before irradiating, to ensure accurate target localization with registrations < 1 mm. After the completion of coplanar beams and the start of non-coplanar beams, a set of CBCT projections for image registration was again administered. The entire procedure was overseen by a radiation oncologist.

### Assessment of outcomes and adverse events

After radiation therapy, the patients visited the outpatient clinic at weeks 1 and 4 after radioablation, and every 1 month for unstable patients and every 3 months for relatively stable patients thereafter. Chest radiography and transthoracic echocardiography were performed to evaluate for possible complications, including pericardial effusion or radiation pneumonitis. Post-radiation MRI or CT evaluations were performed in eligible patients. ICD interrogation was performed at the electrophysiologist visit. For patients with PVCs, 24-h Holter monitoring was performed to assess the radiation efficacy. Anti-arrhythmic medications were titrated by an electrophysiologist according to each patient’s clinical condition.

We analyzed the time to recurrence of ICD therapy after a 6-week blanking period and the time to major events including mortality. The mean numbers of VT episodes (VT burden) and ICD therapies before and after radioablation were also recorded. Acute and late toxicities were rated according to the Common Terminology Criteria for Adverse Events version 4.0.

### Ethical approval

The study was approved by the institutional review board of the National Taiwan University Hospital Ethics Committee. The research was performed in accordance with relevant guidelines/regulations.

### Informed consent

Informed consent was obtained from all patients and/or their legal guardians.

## Results

### Patient population

From February 2019 to December 2019, seven patients were enrolled. Table 1 outlines the clinical characteristics of the patients. Six male and one female patient underwent SBRT. The mean patient age was 55 years (range 23–80 years). Among the seven patients, three had DCM, one had ICM, one had HCM, one had ARVC, and one (patient 3) had frequent PVCs with normal left ventricular ejection fraction (LVEF). The patient had delayed enhancement at the basal anteroseptum of the left ventricle, which was unlikely to have been caused by two previous ablation procedures. The median number of previous catheter ablations before enrollment was 2 (range 0–4). One patient (patient 2) did not undergo catheter ablation therapy owing to left ventricular apical thrombus. Six patients were taking amiodarone, four of whom at a dose of > 300 mg daily, before enrollment.

**Table 1:** Patients’ clinical characteristics and treatment details.

Variable	Patient 1	Patient 2	Patient 3	Patient 4	Patient 5	Patient 6	Patient 7
**Clinical characteristics**							
Age (years)	58	56	49	47	23	72	80
Sex	Female	Male	Male	Male	Male	Male	Male
Indication	Ischemic cardiomyopathy	Dilated cardiomyopathy	Frequent PVCs	Dilated cardiomyopathy	Arrhythmogenic right ventricular cardiomyopathy	Hypertrophic cardiomyopathy	Dilated cardiomyopathy
LVEF before treatment (%)	45	20	66	30	69	42	43
Anti-arrhythmic drugs	Amiodarone	BB + amiodarone	BB + mexiletine	BB + amiodarone	BB + amiodarone	BB + amiodarone	BB + amiodarone
Amiodarone dosage (mg/d)	300	400	0	100	400	400	200
No. of catheter ablations	1	0	2	1	4	2	2
No. of episodes of VT at 3 months before treatment	34	14	PVC 35%	3	6	3	31
No. of ICD shocks	5	3	–	1	6	2	8
**Treatment**							
Target region	Inferoposterior wall	Anterior wall	Basal anteroseptum	RVOT	RV basal septum	Apex	Anteroseptum to inferoseptum
Volume (cm^3^)							
ITV	15.4	40.9	9.4	4.6	11.5	35.3	33.5
PTV	52.1	80.5	23.9	14.4	34.4	83.4	92.6
Time (min)	12.7	17.3	14.4	9.2	10.9	12.5	12.5
No. of episodes of VT after treatment	9	1	PVC 0.2%	0	13	5	0
No. of ICD shocks after treatment	1	0	–	0	0	0	0
Time to recurrence (days)	245	366	–	0	105		
LVEF after treatment (%)	33	22	72	68	65	42	33

*PVC* premature ventricular contraction, *BB* beta-blocker, *LVEF* left ventricular ejection fraction, *VT* ventricular tachycardia, *ICD* implantable cardioverter-defibrillator, *RVOT* right ventricular outflow tract, *RV* right ventricular, *ITV* internal target volume, *PTV* planning target volume.

### Radiation procedure

The SBRT dosimetry and treatment details are shown in Table 2. The median ITV was 11.5 cm^3^ (range 4.6–64.9 cm^3^). Accounting for motion and conservative additional margins for setup and delivery, the median PTV was 34.4 cm^3^ (range 14.4–84.5 cm^3^). In these 7 patients, 3 patients had PTV more than 80 cc, including patient 6 with HCM, and patient 2 and 7 with DCM. A median of 10 partial arcs (range 10–13) were used for the cardiac radioablation plan, all with 4 non-coplanar partial arcs and a median of 6 coplanar partial arcs (range 6–9). All plans met the criteria with appropriate target volume coverage and OAR constraints. The median maximal dose within the ITV was 31.0 Gy (124% of the prescribed dose of 25 Gy), and the minimal dose to cover 95% of the ITV and PTV was at least 25 and 20 Gy, respectively. Efforts were made to minimize radiation delivery to non-target regions, and the median heart volume excluding the PTV (heart minus PTV) receiving at least 16 Gy was only 12.7 cm^3^, which is less than the criterion of 15 cm^3^. The median total monitor units were 8,898 (range 6431–12,092), and the median beam-on time was 12.7 min (range 9.2–17.3 min). No patient required sedation during the procedure. Supplementary Figure S1 shows the SBRT simulation images of all patients.

**Table 2:** Stereotactic body radiotherapy dosimetry and treatment details.

**Median target volume, cm**^**3**^** (range)**	
Internal target volume (ITV)	15.4 (4.6–40.9)
Planning target volume (PTV)	52.0 (14.4–92.6)
**Dosimetry analysis, median (range)**	
ITV, DMax (Gy)	31.0 (29.7–32.3)
ITV, D98% (Gy)	24.8 (24.4–24.9)
ITV, D95% (Gy)	25.1 (25.0–25.5)
ITV, D1% (Gy)	30.2 (29.2–30.9)
PTV, D98% (Gy)	19.4 (19.0–19.7)
PTV, D95% (Gy)	20.1 (20.0–20.7)
PTV, D1% (Gy)	29.7 (29.0–30.3)
Skin, Dmax (Gy)	8.6 (4.9–13.9)
Rib, Dmax (Gy)	13.3 (5.8–25.5)
Trachea and large bronchus, Dmax (Gy)	1.4 (0.3–12.2)
Spinal cord, Dmax (Gy)	2.1 (1.3–4.6)
Stomach, Dmax (Gy)	3.4 (0.1–12.0)
Esophagus, Dmax (Gy)	5.2 (2.7–14.9)
Coronary arteries, Dmax (Gy)	19.0 (8.4–21.8)
Heart minus PTV, > 16.0 Gy (cm^3^)	12.7 (7.7–14.9)
Lung, < 7.0 Gy (cm^3^)	2403 (2041–3739)
Liver, < 9.1 Gy (cm^3^)	1120 (954–1828)
**SBRT energy, n (%)**	
6 MV FFF	2 (28.5)
10 MV FFF	5 (71.5)
**Median SBRT employed partial arcs, number (range)**	
Total arcs	10 (9–13)
Coplanar arcs	6 (5–9)
Non-coplanar arcs	4 (4–4)
Median SBRT monitor units, MU (range)	8898 (6431–12,092)
Median SBRT treatment time, min (range)	12.7 (9.2–17.3)

*SBRT* stereotactic body radiotherapy, *FFF* flattening filter-free photon beam, *Dmax* maximal dose within the volume, *D98%, D95%, and D1%* minimum coverage dose of 98%, 95%, and 1% of the target volumes, respectively, *Heart minus PTV*, heart volume excluding the PTV; > 16.0 Gy, volume receiving at least 16 Gy; < 7.0 Gy and < 9.1 Gy, volumes receiving < 7 Gy and 9.1 Gy, respectively.

### Efficacy

Patient 6 (hepatitis C virus [HCV] carrier) died of hepatic failure on post-treatment day 47. In the remaining six patients, at a median follow-up of 14.5 months (range 10–20 months), the VA burden significantly decreased after treatment (Table 1). Three months before treatment, there were 88 episodes of VTs and 23 shock therapies. After the blanking period (6 weeks after SBRT), there were 23 episodes of VTs during the next 91 patient-months. Only one patient received one shock therapy. Patient 1 with ICM had 34 episodes of VTs and 5 ICD shocks at 3 months before SBRT. Recurrent VA developed on post-treatment day 245. ICD interrogation showed 8 episodes of VTs, which were all terminated after one ATP treatment, and a PVC-induced ventricular fibrillation with successful shock therapy, which was not the previous clinical VT. LVEF decreased after VT recurrence (45–33%). Patient 2 with DCM had 14 episodes of VTs and 3 ICD shocks at 3 months before SBRT. VT recurred on post-treatment day 366 with presentation of chest pain. Standard ECG showed slow VT with a heart rate of 96 bpm, which was also not the previous clinical VT. No ICD therapy was delivered to this patient because the VT cycle length was out of the VT zones of ICD programming. Patient 4 with DCM showed a relevant clinical response. Before treatment, 6,980 episodes of non-sustained VTs at 3 months before treatment were recorded by the ICD. After treatment, only 391 episodes of non-sustained VTs were recorded and every episode was < 1 s. LVEF improved at 6 months after treatment (30–68%). Patient 5 with ARVC had 6 episodes of VTs, all requiring shock therapy, at 3 months before SBRT. The amiodarone dosage was immediately reduced after SBRT. Recurrent VTs occurred on post-treatment day 105. All episodes were < 10 s, and no ICD shock was required. No more VTs were detected 8 months after SBRT. Patient 7 with DCM also had a relevant clinical response. This patient had 31 episodes of VTs and 8 ICD shocks at 3 months before SBRT. No more VT was recorded by the ICD after treatment. For the patient with frequent PVCs (patient 3), the baseline PVC burden was 35%. The burden remained similar (35%) at 6 weeks after SBRT and increased to 44% at 4 months, but decreased to 9% at 7 months and to 0.2% at 12 months of follow-up. Three patients with large PTV (more than 80 cc) all demonstrated good short-term response to SBRT.

### Imaging comparisons

**Figure 3:**
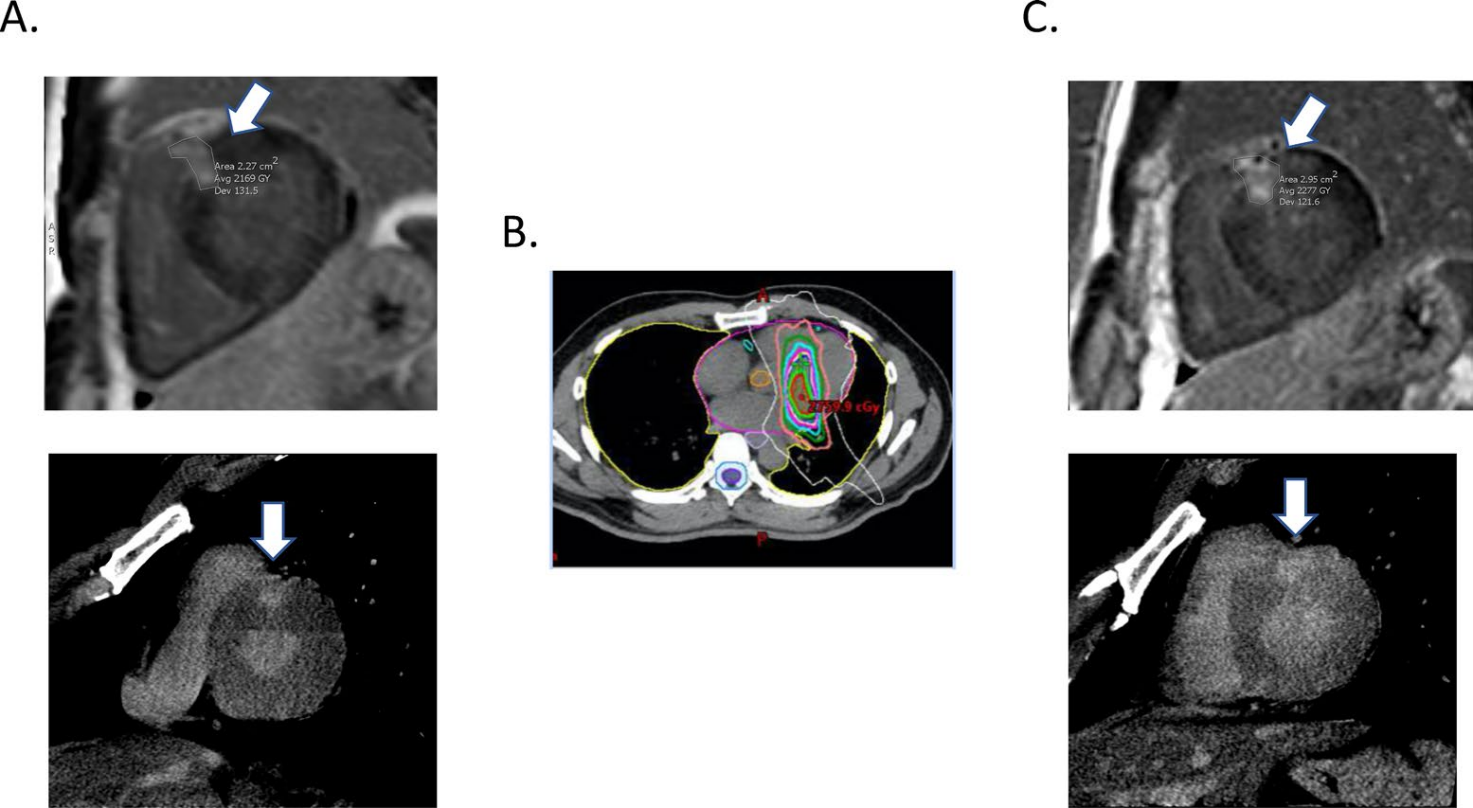
Pre- and post- treatment imaging comparison. (**A**) Pre-treatment CMR (upper part) and dual-energy CT (lower part) of patient 3 showed delayed enhancement of basal antero-septum (white arrow). (**B**) SBRT contour of patient 3. (**C**) Post-treatment CMR (upper part) and dual-energy CT (lower part) of patient 3 showed mildly increased delayed enhancement of basal antero-septum (white arrow). Pre-treatment scar area was 2.27 cm^2^, and post-treatment scar area was 2.95 cm^2^. The radioablation dose distribution figure was created by using treatment planning system (version 15.6, Eclipse, Varian Medical Systems, Palo Alto, URL: http://www.MyVarian.com).

Four patients (patients 3, 4, 5, and 7) underwent pre- and post-treatment CMR imaging. The others did not undergo CMR imaging because their ICDs were not MRI compatible. Patients who could not undergo MRI underwent pre- and post-treatment dual-energy CT. Patient 6 did not have a post-treatment imaging scan because of mortality. The median time of post-treatment imaging was 7 months after SBRT (range 3–12 months). All pre- and post-treatment images are shown in Supplementary Fig. S2. For CMR imaging, the SBRT effects were obvious only in patients 3 and 7, who showed increased scar areas in the post-treatment images compared with the pre-treatment images. For example, the scar area increased from 2.27 cm^2^ in the pre-treatment image to 2.95 cm^2^ in post-treatment image in patient 3 (Fig. 3). In the other two patients (patients 4 and 5), the effects were less obvious. In patient 4, the patient with DCM, pre-treatment CMR imaging and dual-energy CT did not show obvious scarring. The contoured target volume was created by the electrophysiologist according to the 3D electroanatomic map. Only a 4.6 cm^3^ ITV and a 14.4 cm^3^ PTV were irradiated, and the post-treatment images also did not identify scarring. In patient 5, the patient with ARVC, pre-treatment CMR imaging showed diffuse delayed enhancement at the right ventricular outflow tract, free wall, and inferior right ventricular myocardium, and patchy delayed enhancement over the right ventricular basal septum. According to the previous 3D electroanatomic mapping, the SBRT target was the right ventricular basal septum. After treatment, no scar enlargement was noted. Dual-energy CT seemed to be less sensitive in detecting the effect. Only patient 3 had an obvious SBRT effect, but the scar contour was not clear.

### Adverse events

No immediate complications occurred during treatment or hospitalization. All patients were discharged the day after treatment. No ICD dysfunction, including in terms of battery longevity, lead thresholds, or lead impedances, was observed after treatment. Grade 1 pericardial effusion was observed in patient 2 at approximately 6 months after SBRT. Grade 5 hepatic failure occurred in patient 6, which was unlikely to have been caused by radiation therapy.

## Discussion

The chronic therapy of patients with sustained VA includes ICD, anti-arrhythmic drugs, catheter ablation, and/or surgery. For patients with recurrent VA refractory to anti-arrhythmic medication therapy, the current guidelines recommend radiofrequency catheter ablation. A systematic review and meta-analysis of non-randomized trials reported significantly lower rates of recurrent VT after combined endocardial/epicardial ablation than after endocardial ablation alone^13^. Therefore, comprehensive and extensive substrate elimination is the best catheter ablation strategy for recurrent VT. However, some factors might lead to failure of catheter ablation, such as the inability to accurately identify the VT substrate, an extensive substrate not amenable to ablation, or inaccessible VT substrates, such as mid-myocardial, left ventricular summit, or intraseptal areas. In patients with failed catheter ablation, SBRT is an emerging treatment under investigation.

To our knowledge, this is the first reported case series in the Asia–Pacific, which included seven patients with five different VA etiologies. This case series is also the first to compare pre-treatment and post-treatment imaging data.

With respect to SBRT, compared with previously published studies, we used the SIB technique to deliver 25 Gy to the ITV and 20 Gy to the PTV in a single fraction, whereas other studies prescribed a single dose of 25 Gy to the PTV^6,7,14^. Our clinical results demonstrated that this strategy in Asia–Pacific patients for treating refractory VT is as effective as that used in previous studies in Western population. Reduced PTV dose may clinically be safer due to less high irradiation exposure to normal organs, based on accurate respiratory management to lessen lung movements; whether reduced PTV dose potentially influence long-term durable response may need additional time for efficacy evaluation. In the present study reporting the first seven patients, we limited diaphragmatic motion through external abdominal compression per the ENCORE study protocol^7^; we implemented short duration of a median of eight days (range 7–10 days) from CT simulation to treatment in order to minimize discordance of breath patterns between simulation and treatment, as compared to a relative long duration of 13 days by ENCORE study data^15^. For the purpose of minimizing complexity of evaluating both respiratory and cardiac motions, accurately controlling respiratory motion as much as possible is necessary^16^, therefore precise respiratory gating by Respiratory Gating for Scanners (RGSC, phase gating on 30–70% treatment phases) has been applied for further enrolled patients, in order to achieve higher dynamic treatment precision.

In all previously reported case series^6,7,14,17,18^, VT episodes significantly decreased after the 6-week blanking period. In our study, VT episodes decreased by 91% and ICD shock therapy decreased by 86%. All patients responded to SBRT. In addition, all patients with VT showed acute effects of SBRT with no recurrence in the blanking period. However, more patients with different cardiomyopathies should be enrolled to evaluate the possible different SBRT response to each etiology.

In the ENCORE VT study, two patients with PVC-related cardiomyopathy were enrolled, one of whom showed increased PVC burden at 6 weeks, which decreased at the 3-month follow-up. In patient 3 in the present study, the PVC burden was stable at 6 weeks, increased at 4 months, and decreased at 7 and 12 months. The effects of SBRT on PVCs were more delayed than those in the previous study. As the response of PVC burden to radioablation has been less reported, further studies are needed to clarify the time course of response after SBRT.

Previous studies have demonstrated the radiobiological mechanisms of SBRT. Recent evidence indicates that SBRT causes direct cell death due to DNA damage and indirect cell death through vascular damage^19^. Garcia-Barros et al.^20^ reported that irradiation of tumors at doses of > 8–10 Gy rapidly caused ceramide-mediated apoptotic death in endothelial cells, thereby leading to vascular occlusion and tumor cell death. Cuculich et al.^6^ demonstrated the first post-mortem cardiac samples at 3 weeks after SBRT, which showed prominent ectatic blood vessels at the interface of the dense scar and viable myocardium (scar border zone). The present study is the first to compare pre- and post-SBRT images. The images of most of the patients showed denser or more extensive scars after SBRT, as expected. In patient 4, both pre- and post-treatment images did not show obvious scars. This patient underwent SBRT with only a 4.6 cm^3^ ITV and a 14.4 cm^3^ PTV. Although CMR imaging could detect a myocardial scar as small as 1 cm^3^, it is probable that the biological effects of radiation exposure cannot be precisely predicted, and the scar might have been too small to be detected by CMR imaging in this patient. In patient 5, the post-treatment scar at the right ventricular basal septum was stable (without enlargement). The radiation effects may be correlated with the nature of exposure and its extent, as well as the microenvironment of the target tissues. Whether the septum (thicker myocardium) is more radioresistant and requires higher doses is unknown.

No severe acute adverse events were reported in previous studies^6,7,14,17,18^. Radiation pneumonitis and pericardial effusions were the most commonly reported clinically relevant adverse events. In our study, no radiation pneumonitis was detected by post-treatment chest radiography and CT. In patient 2, the post-treatment CT at 6 months after SBRT revealed grade 1 pericardial effusion, which was absent in the previous follow-up transthoracic echocardiography. The pericardial effusion persisted in the latest transthoracic echocardiography follow-up.

Patient 6 died of hepatic failure. He was an HCV carrier without a previous follow-up history. His baseline liver function was normal. One month after SBRT, he visited the emergency department because of progressive jaundice and dyspnea. The HCV viral load was 205,000 IU/mL. The ITV of this patient was 35.5 cm^3^ and the PTV was 83.4 cm^3^. The mean dose for the liver was 0.4 Gy, and a < 1 cm^3^ liver volume received > 9.1 Gy per single fraction, which is considered a very low radiation dose to the liver. Although a certain amount of excess radiation could have been delivered owing to radiation delivery uncertainties, including with respect to positioning and heart and respiratory movements, considering that the target lesion located at the left inferior portion of the heart was not attached to the liver and efforts were made to minimize radiation dose to non-target regions, radiation-related hepatic failure was therefore considered unlikely. Heart failure with comorbid HCV infection was proposed to be related to the hepatic failure in this patient. In the two largest reports^7,14^, most of the mortality cases were related to heart failure and none were related to radiation. Nevertheless, more advanced planning techniques, including robust optimization, should be considered for achieving the most precise radiation delivery^21^. Although our current version of the TPS (Eclipse; Varian Medical Systems, Palo Alto, CA, USA) does not support robust treatment planning optimization, our institutional license in the near future will include this advanced function.

Before large-scale long-term follow-up data emerge, SBRT may be an alternative treatment for patients with life-threatening or severely symptomatic VAs that are refractory or ineligible to traditional treatments. As long as the OAR constraints for SBRT set by the AAPM TG101 are cautiously satisfied^9^, long-term complications could be minimized in patients undergoing SBRT.

### Limitations

This was a small, single-center, retrospective analysis with a limited follow-up time. The long-term efficacy and safety of this treatment is still unknown.

## Conclusion

In conclusion, in patients with medication- and catheter ablation–refractory VT of variable etiologies, SBRT for cardiac radioablation is associated with a marked reduction in the burden of VA.

## Supplementary Information

Supplementary Figure S1.

Supplementary Figure S2.
