# Supplementary material for: First Asian population study of stereotactic body radiation therapy for ventricular arrhythmias
Source: Sci Rep. 2021 May 14;11:10360. doi: 10.1038/s41598-021-89857-2 (PMC8121933; doi:10.1038/s41598-021-89857-2)
Supplement: Supplementary file 1 — Supplementary Figure S1. [file 41598_2021_89857_MOESM1_ESM.pdf]

Supplement 1. SBRT simulation images of all patients.

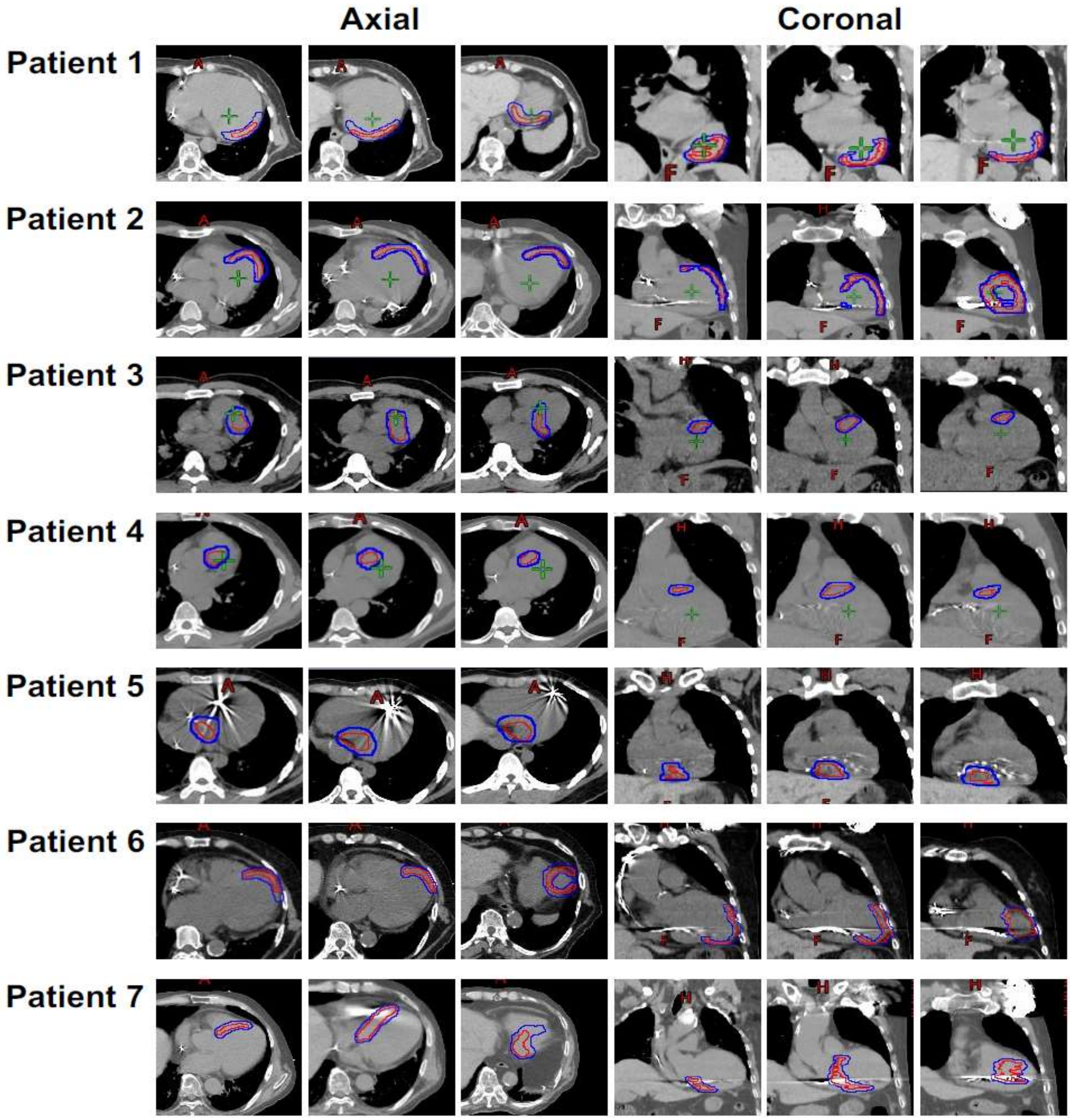

The red lines indicate the gross target areas and the blue lines indicate the planning target areas. Radiation targets are illustrated in the axial and coronal views (the figures were created by treatment planning system, version 15.6, Eclipse, Varian Medical Systems, Palo Alto, URL:http://www.MyVarian.com)
