# Supplementary material for: First Asian population study of stereotactic body radiation therapy for ventricular arrhythmias
Source: Sci Rep. 2021 May 14;11:10360. doi: 10.1038/s41598-021-89857-2 (PMC8121933; doi:10.1038/s41598-021-89857-2)
Supplement: Supplementary file 2 — Supplementary Figure S2. [file 41598_2021_89857_MOESM2_ESM.pdf]

Supplement 2. Pre- and post-SBRT images in all patients

Pre-SBRT CT Post-SBRT CT

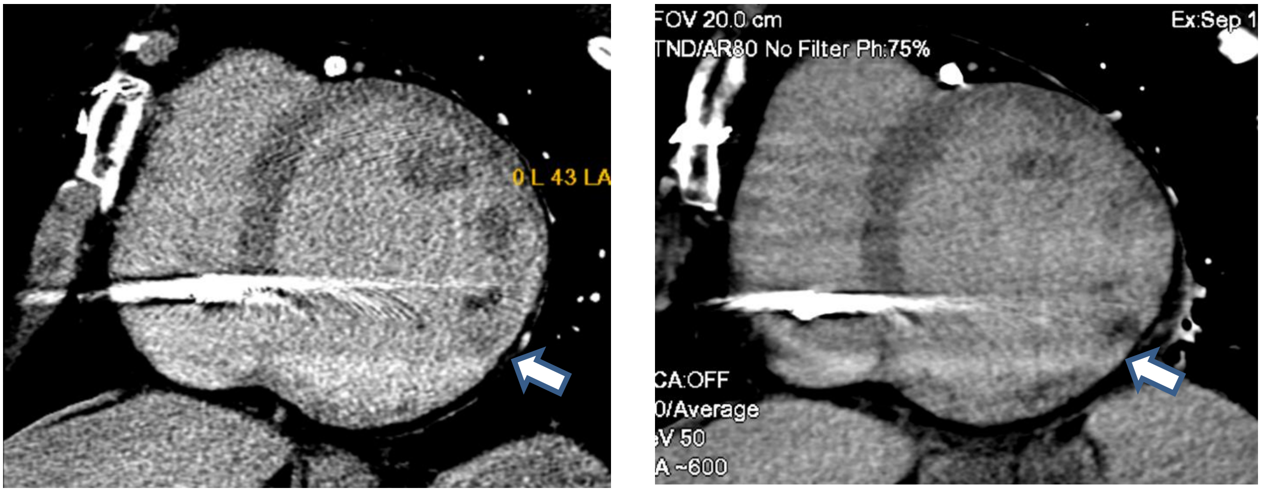

2.1 Pre- and post-SBRT CT in patient 1. Scar at infero-posterior wall increased after treatment.

Pre-SBRT CT Post-SBRT CT

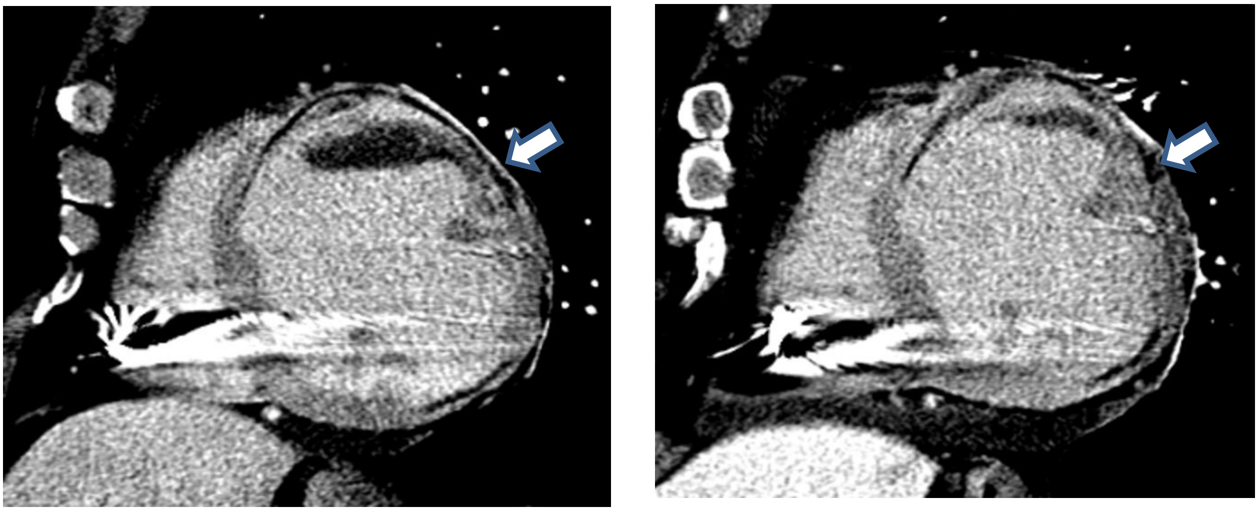

2.2 Pre- and post-SBRT CT in patient 2. Scar at anterior wall increased after treatment. LV thombus decreased in size.

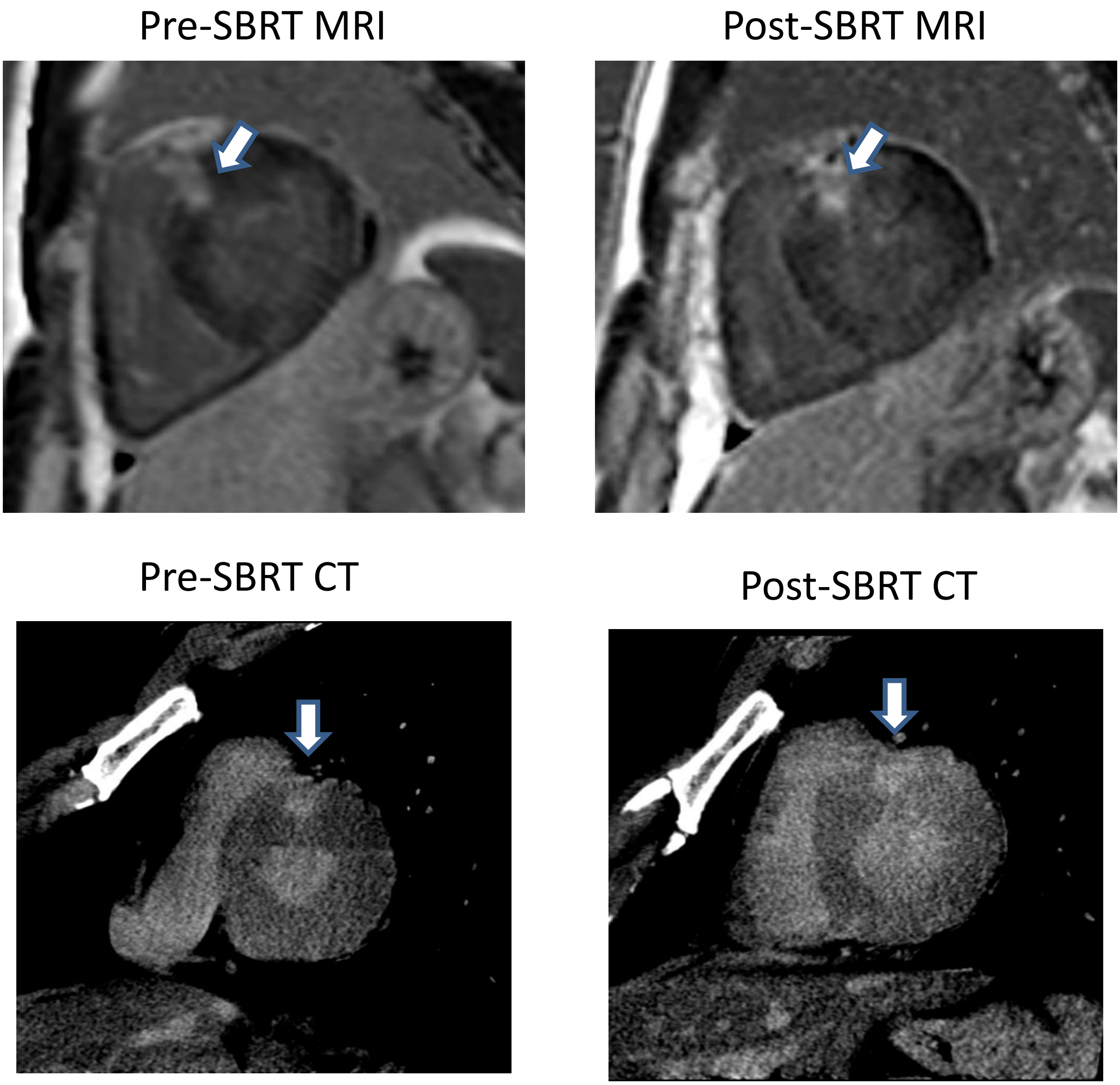

2.3 Pre- and post-SBRT MRI (upper part) and CT (lower part) in patient 3. Scar at basal antero-septum increased after treatment.

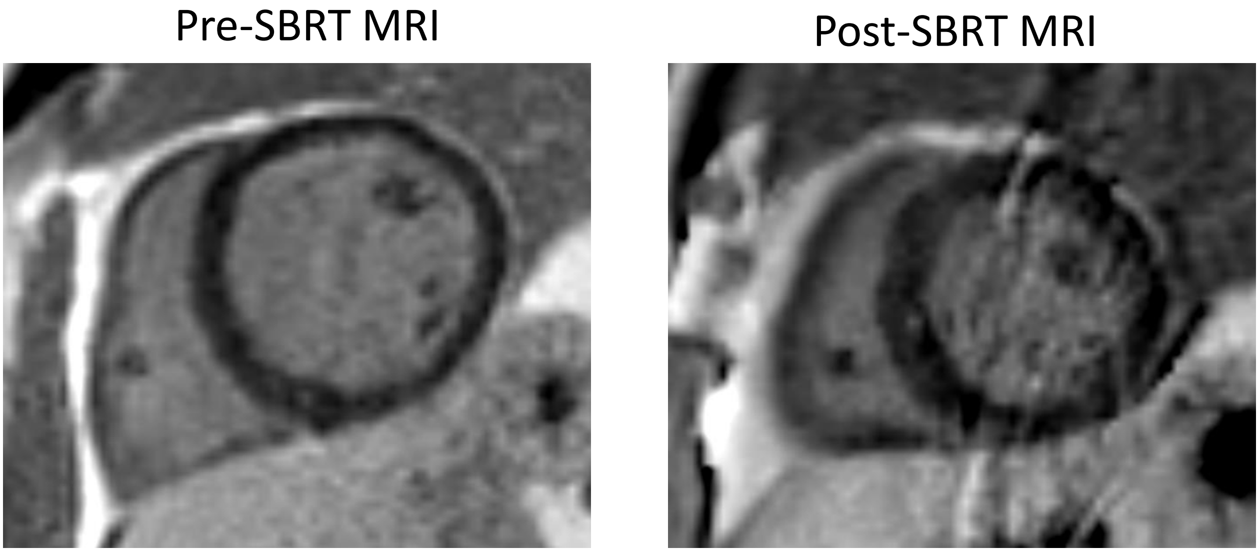

2.4 Pre- and post-SBRT MRI in patient 4. No scar was identified on either pre- and post-SBRT MRI.

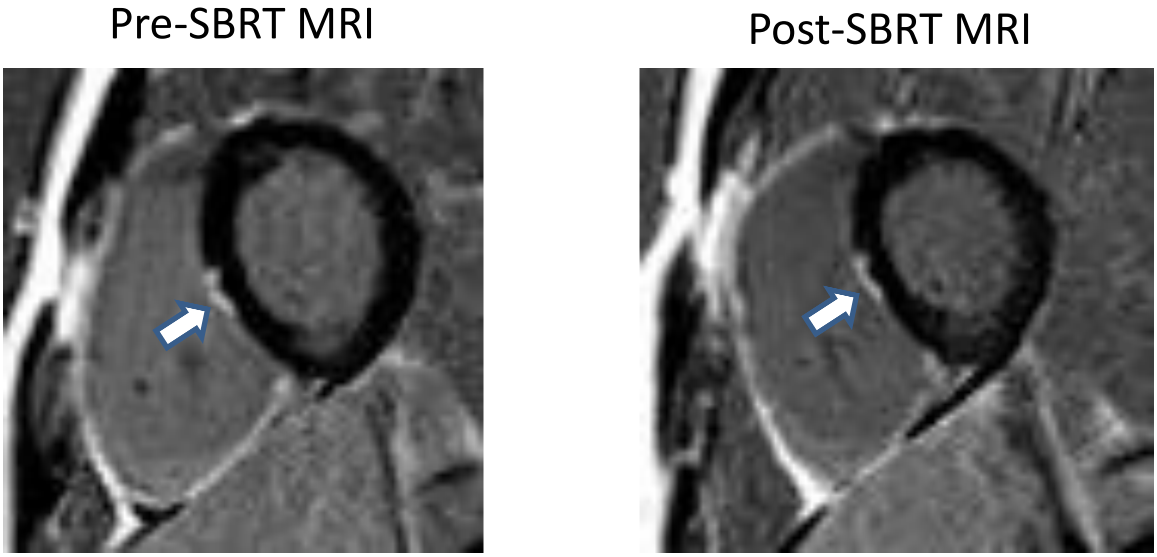

2.5 Pre- and post-SBRT MRI in patient 5. No scar enlargement over RV basal septum (SBRT target) was noted.

Pre-SBRT MRI Post-SBRT MRI

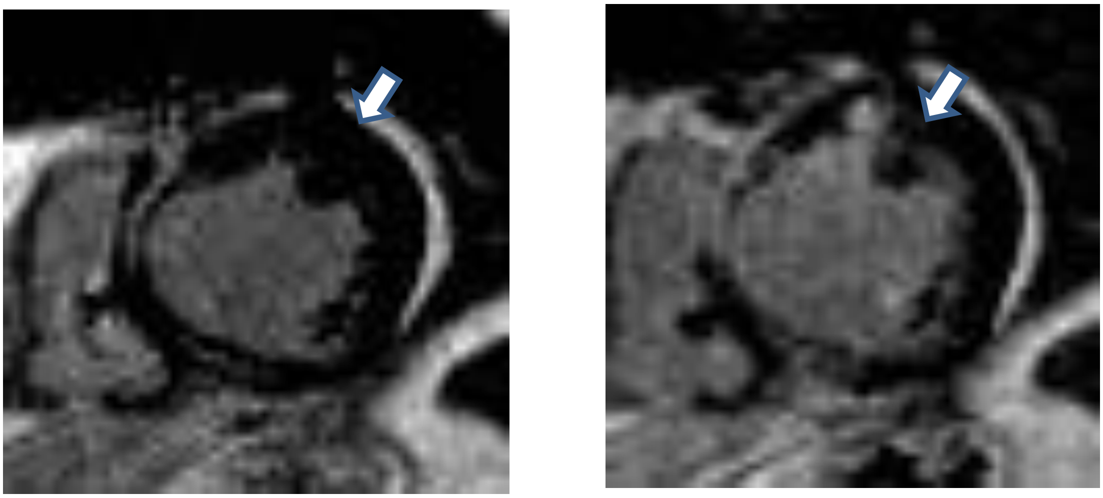

2.6 Pre- and post-SBRT MRI in patient 7. Scar at antero- to infero-septum increased after treatment.
